# Matrix metalloproteinases in the cervical mucus plug in relation to gestational age, plug compartment, and preterm labor

**DOI:** 10.1186/1477-7827-8-113

**Published:** 2010-09-24

**Authors:** Naja Becher, Merete Hein, Carl C Danielsen, Niels Uldbjerg

**Affiliations:** 1 Department of Obstetrics and Gynecology, Aarhus University Hospital Skejby, DK-8200 Aarhus N, Denmark; 2 Department of Connective Tissue Biology, Institute of Anatomy, University of Aarhus, DK-8000 Aarhus C, Denmark

## Abstract

**Background:**

High concentrations of matrix metalloproteinases (MMPs) and tissue inhibitors of metalloproteinases (TIMPs) have been identified in the cervical mucus plug (CMP) at term of pregnancy. Their physiological and pathophysiological implications, however, remain to be elucidated, and CMPs from preterm labor have never been examined. This study was therefore conducted to describe the concentrations of MMP-2, TIMP-1, MMP-8 and MMP-9 in the CMP in relation to gestational age, IL-8 as an indicator of inflammation, compartment of the CMP, and preterm labor.

**Methods:**

An aliquot of the distal plug compartment facing the vaginal microflora (CMP-dist) was collected from non-pregnant (n = 15), early pregnant (n = 15) and term pregnant women (n = 15). Whole CMPs shed during active vaginal term (n = 15) and preterm (n = 4) labor were also included. Protein concentrations were determined by enzyme-linked immunosorbent assay (ELISA).

**Results:**

MMP-2 was not detectable in the non-pregnant CMP-dists whereas high concentrations were found in early pregnancy followed by an 85% decline at term. High concentrations of TIMP-1 were found in both the non-pregnant and early pregnant CMP-dists with a 90% decline at term. Consequently, the molar TIMP/MMP ratio was 40 in the non-pregnant state and 0.2 at term. The MMP-2 and TIMP-1 concentrations were alike in the CMP-dists and the whole CMPs.

MMP-8, MMP-9, and IL-8 were mainly found in the distal CMP compartment. MMP-8 and MMP-9 concentrations were several fold increased in this compartment during pregnancy compared to the non-pregnant state. In the preterm whole CMPs, MMP-8, MMP-9 and IL-8 were 2 to 5 fold increased compared to term whole CMPs.

**Conclusions:**

These results suggest that CMP MMP-2 reflects the non-leukocyte dependent cervical remodeling that occurs in early pregnancy, whereas MMP-8 and MMP-9 are involved in the defense against ascending infections primarily located to the distal compartment of the CMP. The upregulation of MMP-8, MMP-9 and IL-8 in whole CMPs from preterm labor may indicate the involvement of an intrauterine infection.

## Background

**Figure 1:**
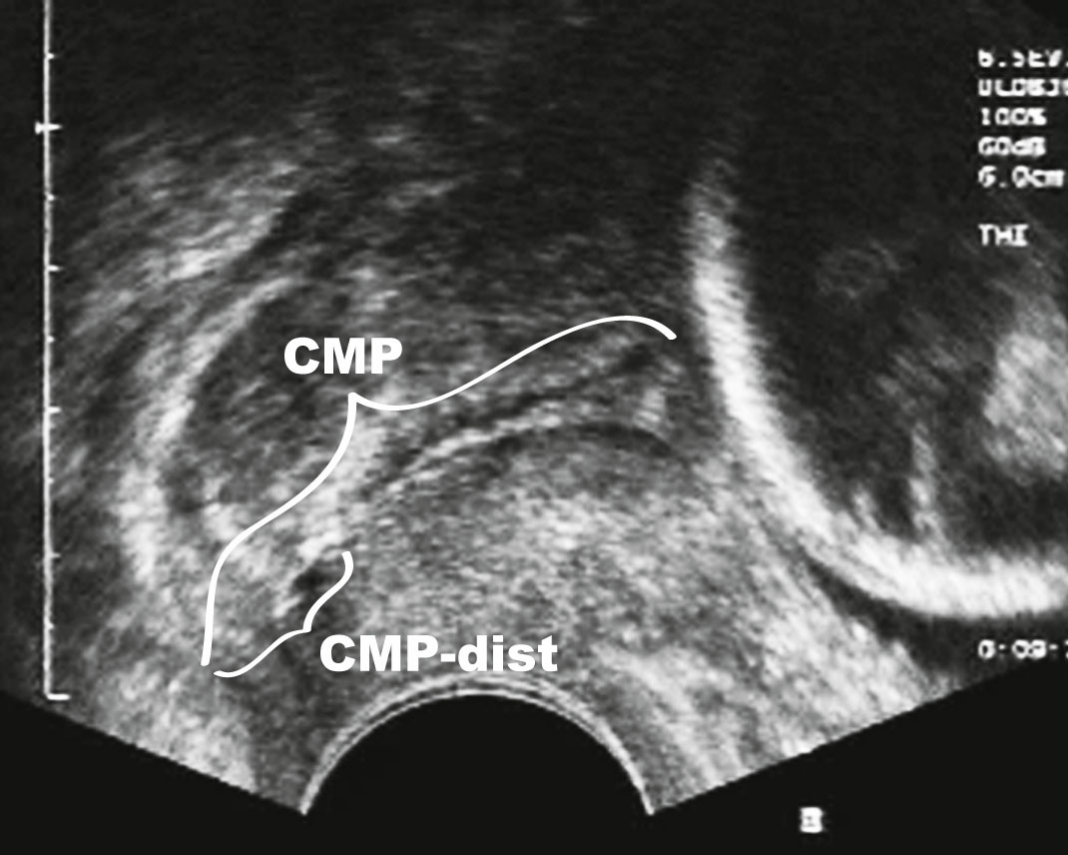
**Definition of the CMP-dist**. Ultrasonography showing the CMP in the cervical canal of a pregnant woman (gestational age 22 weeks). CMP-dist is defined as the part of the plug closest to the vaginal microflora.

Preterm delivery accounts for over one million newborn deaths worldwide each year [1], and it is widely recognized that ascending intrauterine infection is to blame in 25-40% of these cases [2-4]. In normal pregnancies, the cervical mucus plug (CMP) provides a protective barrier against ascending infection by possessing strong antibacterial properties and significant innate and adaptive immune functions [5,6]. The CMP fills the cervical canal (Figure 1) and is distinguishable from cervico-vaginal fluid because of its unique viscoelastic appearance. When shed during labor, it is a large, well-defined structure, of approximately 10 grams [7].

Matrix metalloproteinases (MMPs) are proteolytic enzymes with the potential to degrade extracellular matrix components [8] and to activate cytokines and anti-microbial peptides [9]. In obstetrics, MMPs are implicated in cervical dilatation [10], fetal membrane rupture [11] and in the pathology of preterm delivery [12]. Particularly elevated amniotic fluid concentration of MMP-8 has attracted attention as an indicator of intrauterine infection [13].

MMP-2, MMP-9 and MMP-8, together with their specific inhibitors, the tissue inhibitors of metalloproteinases (TIMPs), have been quantified in the CMPs from women in active labor at term [14,15]. However, our knowledge of the physiological and pathophysiological roles of MMP and TIMP in the CMP is still limited, and their concentrations in CMPs from preterm labor remain to be described. We do not know whether CMP MMPs reflect MMP activity in the cervical stroma and in the fetal membranes, or whether they are involved in processes within the CMP, e.g. local inflammatory processes.

This study was conducted to evaluate the concentrations of MMP-2, TIMP-1, MMP-8, and MMP-9 in the CMP in relation to gestational age, IL-8 as an indicator of inflammation, compartment of the CMP, and preterm labor.

## Methods

Two subsets of CMP samples were included in this study: distal CMP samples and whole CMP samples. These samples were collected from two separate populations.

### Sample collection

*Distal CMP samples *were collected from the distal compartment of the plugs, i.e. the part closest to the vaginal microflora, referred to as *CMP-dists *(Figure 1). Collection of CMP-dist aliquots was performed with a 1-ml syringe, a peang, or a forceps during visual inspection of the cervix. The CMP-dist aliquots consisted of pure CMP material, but minor surface contamination with vaginal fluid and/or blood could not be completely avoided in all cases.

The *non-pregnant *group included 15 clomiphene/FSH stimulated women attending the Fertility Clinic for in vitro fertilization on the day of oocyte aspiration. The *early pregnant *group included 15 women having an elective termination of pregnancy (gestational age (GA) 7-12 weeks). The *term pregnant *group included 15 women undergoing either an elective cesarean section or a labor induction (GA 37-42 weeks).

*Whole CMPs *were collected during routine vaginal exploration or shed spontaneously (cervical dilatation 2-10 cm) from women in spontaneous vaginal labor at *term *(n = 15, GA 37-42 weeks) or *preterm *(n = 4, GA 30^+2^, 35^+5^, 36^+0 ^and 36^+4 ^weeks respectively). None of the woman in the preterm labor group had preterm prelabor rupture of the membranes.

The baseline characteristics of the participants are provided in Table 1. None of them had received prostaglandins for pre-induction cervical ripening, glucocoticoids for fetal lung maturation or antibiotics prior to sample collection. Pre-eclamptic women were excluded. The Central Denmark Region Committee on Biomedical Research Ethics approved the project (project ID: 20050053), and written informed consent was obtained from all participants in the study.

**Table 1:** Baseline characteristics and vaginal culture results

Group	n	Age, years median (range)	Gestational age weeks^days^ median (range)	Parity median (range)	Vaginal culture potential pathogens/total	Weight, gram mean (95% CI)
Non-pregnant CMP-dist	15	32 (20-39)	0	0	1/15	0.47 (0.34-0.60)
Early pregnant CMP-dist	15	34 (20-42)	8^+1 ^(6^+4 ^- 11^+0^)	2 (1-4)	3/15	0.39 (0.25-0.52)
Term pregnant CMP-dist	15	32 (20-39)	39^+3 ^(37^+0 ^- 42^+0^)	1 (1-3)	5/15	0.35 (0.081-0.63)
Term labor whole CMP	15	31 (23-36)	40^+0 ^(38^+5 ^- 41^+3^)	1 (0-3)	not performed	7.0 (4.4-9.6)
Preterm labor whole CMP	4	29 (22-32)	36^+0 ^(30^+2 ^- 36^+4^)	0 (0-1)	not performed	3.8 (0.75-6.9)

The weight of the samples are given in the last column. Comparisons performed by *t-test *showed no statistical difference in sample weights between the groups: non-pregnant CMP-dist vs. early pregnant CMP-dist (*P *= 0.4), non-pregnant CMP-dist vs. term pregnant CMP-dist (*P *= 0.4), early pregnant CMP-dist vs. term pregnant CMP-dist (*P *= 0.8), term labor whole CMP vs. preterm labor whole CMP (*P *= 0.2).

### Extraction

All samples were stored at - 80°C until extraction. Initially, they were pulverized in liquid nitrogen with a manual mortar, and 100 mg of this mixture was homogenized manually on ice in 2.9 ml of extraction buffer (50 mM Tris-HCl, 10 mM CaCl_2_, 0.05% Brij 35 and 1 mM phenylmethylsulfonyl fluoride, pH 7.4). From this 1:30 dilution, an aliquot was extracted overnight at 4°C with rotation and thereafter subjected to centrifugation (20 minutes, 16,000 g, first extraction). More buffer was then added to the pellet, which was re-homogenized on ice and re-extracted overnight (second extraction). Finally, a short heat extraction was performed on the pellet (4 min, 60°C, third extraction). Total extraction buffer added was adjusted to a 1:100 dilution. The three supernatants were pooled (total volume 2 ml) and kept at - 80°C until analysis.

### Enzyme-linked immunosorbent assay

The concentrations of MMP-2, TIMP-1, MMP-8, MMP-9 and IL-8 were measured in duplicates by means of commercially available ELISA-kits (GE Healthcare, Buckinghamshire, UK).

The *MMP-2 *kit (product number RPN2617) recognized proMMP-2 and proMMP-2 in complex with TIMP-2 in the range between 1.5 and 24 ng/ml. The interassay coefficient of variation (CV) was 12.2%, and the intra-assay CV was 6.3%. The *TIMP-1 *kit (RPN2611) measured total TIMP-1 (free TIMP-1 and that in complex with MMP-1, 2, 3 and 9) in the range between 3.13 and 50 ng/ml. The interassay CV was less than15.2% and the intra-assay CV less than 11.4%.

*MMP-8 *(RPN2619) was measured in a 0.25-4 ng/ml range and recognized as pro and active MMP-8, both free and in complex with TIMPs. The interassay CV was less than 6% and the intra-assay CV less than 2.9%. *MMP-9 *(RPN2614) was measured as proMMP-9 and proMMP-9/TIMP-1 complexes in the range between 1-32 ng/ml. The interassay CV was less than 9%, and the intra-assay CV less than 5.5%. *IL-8 *(RPN2764) was measured in a range from 26-1000 pg/ml. Both inter and intra-assay CVs were below 10%.

### Vaginal culture

A culture sample from fornix posterior was obtained by a carbon coated cotton wool swab prior to the collection of the CMP-dists (n = 45). The swab samples were cultured on 5% blood agar overnight at 37°C, and bacterial growth was described as the relative number of cultures. Only the presence of Group B or G *Streptococcus*, pure cultures of *Staphylococcus aureus *or overgrowth of Candida albicans were considered as potential pathogens.

### Blood contamination

The hemoglobin (Hb) concentrations were measured in the 15 CMP-dists from the non-pregnant group, the 15 CMP-dists from the early pregnant group and the 15 whole CMPs from the term labor group (KoneLab 30i, Thermo Scientific).

### Statistics

To obtain a normal distribution and an equal variance, the data were log-transformed as appropriate. Non-detectable (ND) was defined as values below the ELISA standard curve, and in these cases the sensitivity of the kit was inserted before log-transformation. When the log-transformed data could not meet the criteria of a parametric test, a non-parametric test was used. One-way analysis of variance (ANOVA) was performed among the non-pregnant, the early pregnant and the term pregnant groups followed by post hoc pairwise multiple comparisons (Holm-Sidak method). We used Student's *t test *when comparing two groups. Linear regression analysis was used to test associations between selected concentrations. Results are given as mean (95% confidence intervals (95% CI)). All statistical analyses were performed using SigmaStat^® ^3.5 (Systat). *P*-values below 0.05 were considered significant.

## Results

### Gestational age (Table 2, Figure 2)

**Table 2:** Gestational age

Group	n	MMP-2 ng/mg CMP	TIMP-1 ng/mg CMP	MMP-8 ng/mg CMP	MMP-9 ng/mg CMP	IL-8 pg/mg CMP	Molar ratio TIMP/MMP
Non pregnant	15	ND	6.3 (3.4-12)	0.020 (0.004-0.10)	0.32 (0.14-0.70)	0.031 (0.004-0.26)	40 (14-110)
Early pregnant	15	3.6 (2.4-5.3)	11 (9.1-14)	3.6 (2.5-5.2)	4.8 (3.5-6.6)	101 (72-139)	2.2 (1.6-3.1)
Term pregnant	15	0.54 (0.23-1.3)	1.2 (0.48-2.8)	6.6 (3.4-13)	7.5 (3.9-15)	213 (139-327)	0.20 (0.071-0.58)
ANOVA *P*-value	45	< 0.001	< 0.001	< 0.001	< 0.001	< 0.001	< 0.001

Concentrations and the molar TIMP/MMP ratios in the CMP-dists from non pregnant, early pregnant and term pregnant women. Protein concentrations are given as geometric mean (95% CI). ND: not detectable.

**Figure 2:**
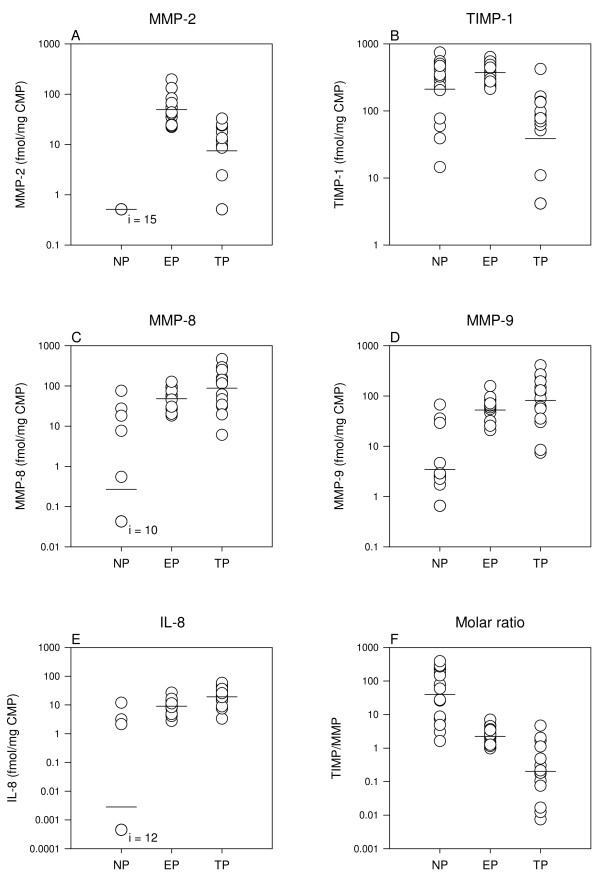
**Gestational age**. One-way analysis of variance showed different mean values in each of the panels A-F (*P *< 0.001). The *P*-values of the post-hoc pairwise comparison are listed in the results section. NP: non-pregnant (n = 15), EP: early pregnant (n = 15), TP: term pregnant (n = 15), i: subset of n. Note the log-scale.

*MMP-2 *was not detectable in the non-pregnant CMP-dists, whereas the mean concentration was 3.6 ng/mg CMP in early pregnancy followed by a decline to 0.54 ng/mg CMP at term (*P *< 0.001) (molar concentrations (fmol/mg CMP) are shown in Figure 2A).

The mean *TIMP-1 *concentration in the early pregnant CMP-dists was unchanged from that found in the non-pregnant CMP-dists (*P *= 0.2) and followed by a decline at term (*P *< 0.001) when using post hoc pairwise comparison (Figure 2B). A subsequent comparison performed by *t-test *showed that TIMP-1 tended to increase from the non-pregnant to the early pregnant state (*P *= 0.08). The highest molar inhibitor/enzyme ratio (TIMP-1/(MMP-2+MMP-8+MMP-9)) of 40 was found in the non-pregnant CMPs (Figure 2F). This ratio decreased to 2.0 in early pregnancy (*P *< 0.001) followed by a further drop to 0.2 at term (*P *< 0.001). CMP-dists from pregnant women containing high concentrations of TIMP-1 also contained high MMP-2 (R = 0.69, *P *< 0.001).

*MMP-8 and MMP-9 *concentrations were positively correlated (R = 0.79, *P *< 0.001) and they both showed a several fold increase from the non-pregnant state to the first trimester of pregnancy (*P *< 0.001) (Figures 2C and 2D). Their concentrations were nearly doubled from early pregnancy to term, but this change did not reach statistical significance (*P *≥ 0.3) by post hoc pairwise comparison. A subsequent *t-test *showed that MMP-8 tended to increase from early pregnancy to term (*P *= 0.1).

*IL-8 *protein concentrations in the CMP-dists demonstrated the same pattern as MMP-8 and MMP-9: low or non-detectable in the non-pregnant state, a rise in early pregnancy (*P *< 0.001) and doubled concentrations at term (*P *= 0.4) (Figure 2E). When comparing early and term pregnant IL-8 in the CMP-dists by *t-test*, this difference was statistically significant (*P *= 0.006). IL-8 protein was positively correlated to MMP-8 (R = 0.68, *P *< 0.001) and MMP-9 (R = 0.45, *P *= 0.001) and negatively correlated to TIMP-1 (R = - 0.30, *P *= 0.04).

### Plug compartment (Table 3, Figure 3)

**Table 3:** Plug compartment

Group	n	MMP-2 ng/mg CMP	TIMP-1 ng/mg CMP	MMP-8 ng/mg CMP	MMP-9 ng/mg CMP	IL-8 pg/mg CMP	Molar ratio TIMP/MMP
Term CMP-dist	15	0.54 (0.23-1.3)	1.2 (0.48-2.8)	6.6 (3.4-13)	7.53 (3.9-15)	213 (139-327)	0.20 (0.071-0.58)
Term whole CMP	15	0.48 (0.16-1.4)	1.4 (0.57-3.5)	1.9 (1.2-2.9)	1.7 (0.88-3.4)	78 (55-110)	0.72 (0.26-2.0)
Student's *t-test* *P*-value		0.9	0.7	0.002	0.003	< 0.001	0.07

Comparison of concentrations and molar TIMP/MMP ratios in the term CMP-dists (from Table 2) and the term whole CMPs (from Table 4) which were collected from two separate populations. Protein concentrations are given as geometric mean (95% CI).

**Figure 3:**
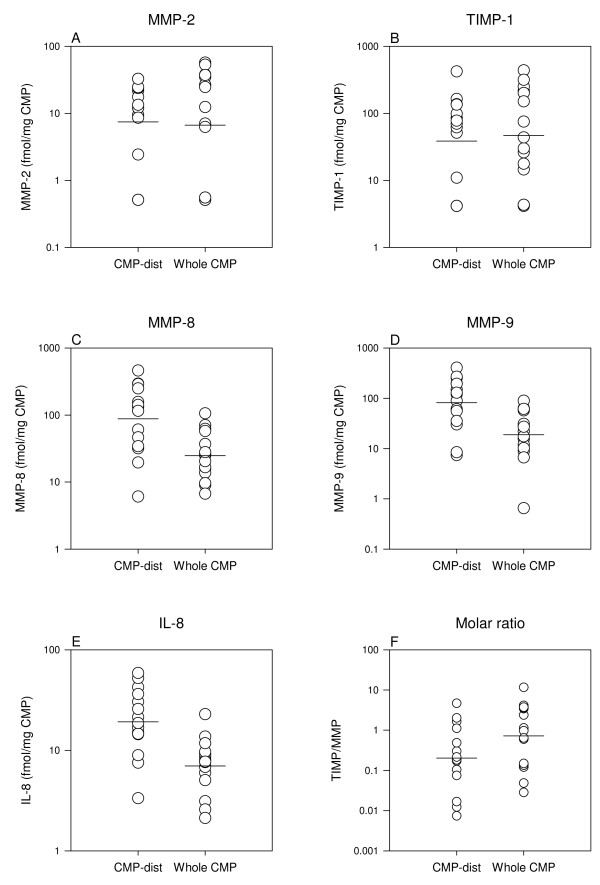
**Plug compartment**. Comparisons of concentrations and the molar TIMP/MMP ratios in the term CMP-dists (n = 15) and the term whole CMPs (n = 15). Results from Students *t-test *are listed in Table 3. Note the log-scale.

Concentrations of MMP-8 (*P *= 0.002), MMP-9 (*P *= 0.003) and IL-8 (*P *< 0.001) were 3-4 fold higher in the 15 term pregnant CMP-dists as compared to the 15 term labor whole CMPs. Concentrations of MMP-2 and TIMP-1 were alike in the two groups (*P *≥ 0.7).

### Preterm and term labor (Table 4, Figure 4)

**Table 4:** Preterm and term labor

Group	n	MMP-2 ng/mg CMP	TIMP-1 ng/mg CMP	MMP-8 ng/mg CMP	MMP-9 ng/mg CMP	IL-8 pg/mg CMP	Molar ratio TIMP/MMP
Preterm labor	4	1.4 (0.40-4.7)	3.3 (0.90-13)	4.7 (0.29-77)	7.6 (0.90-65)	221 (34-1442)	0.58 (0.040-8.8)
Term labor	15	0.48 (0.16-1.4)	1.4 (0.57-3.5)	1.9 (1.2-2.9)	1.7 (0.88-3.4)	78 (55-110)	0.72 (0.26-2.0)
Student's *t test* *P*-value		0.3	0.3	0.1	0.05	0.03	0.8

Comparison of concentrations and molar TIMP/MMP ratios in the preterm and term whole CMPs. Protein concentrations are given as geometric mean (95% CI).

**Figure 4:**
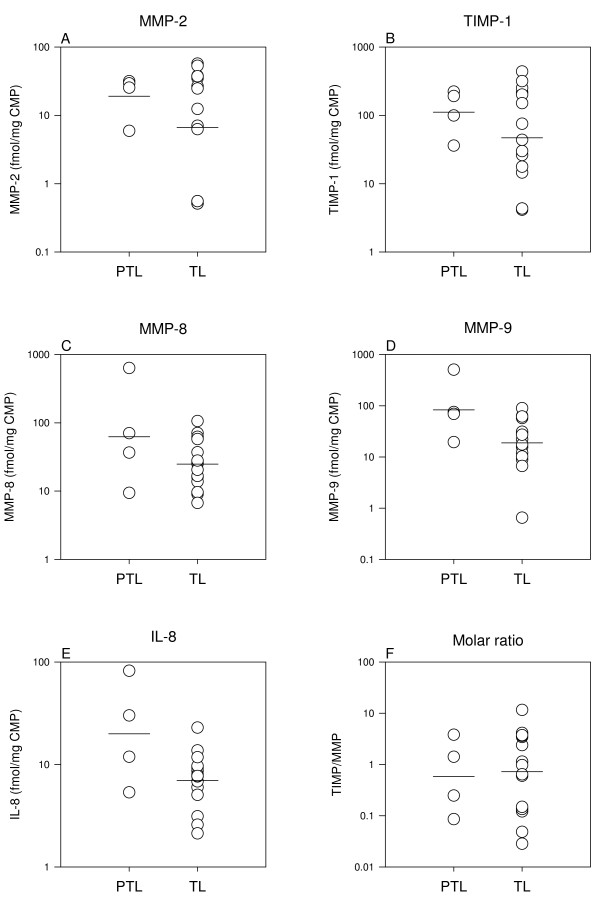
**Preterm and term labor**. Comparison of concentrations and the molar TIMP/MMP ratios in preterm (PTL, n = 4) and term (TL, n = 15) whole CMPs. Results from Students *t-test *are listed in Table 4. Note the log-scale.

MMP-9 (*P *= 0.05) and IL-8 (*P *= 0.03) were 3 to 5 fold higher in the whole CMPs from preterm labor as compared to whole CMPs from term labor. A doubling of MMP-8 failed to reach statistical significance (*P *= 0.1). MMP-2 and TIMP-1 concentrations were also doubled in the preterm plugs but without reaching statistical significance (*P *≥ 0.3). One CMP, collected during very preterm labor (week 30^+2^), contained particularly high concentrations of MMP-8 (48 ng/mg CMP), MMP-9 (47 ng/mg CMP) and IL-8 (911 pg/mg CMP) (Figures 4C, D and 4E).

### Vaginal culture

Microbiological examination demonstrated potential pathogens in 9 of 45 women: Group B *Streptococcus *(6/45), Group G *Streptococcus *(1/45) and overgrowth of Candida albicans (2/45). No pure cultures of *Staphylococcus aureus *were found. MMP-2, TIMP-1, MMP-8, MMP-9 and IL-8 concentrations seemed unaffected in the 9 women with potential pathogens. The distribution of positive vaginal cultures across the groups is evident from Table 1.

### Blood contamination

Assuming a blood hemoglobin (Hb) concentration of 7 mM, the median (interquartile range) concentration of blood in the CMP was ND (0-3%) in the non-pregnant group, 9% (1-11%) in the early pregnant group and ND (0-3%) in the whole CMPs from term labor. There was no association between Hb content and the components measured in the group showing a 9% blood contamination (*P *> 0.3).

## Discussion

The main findings of the present study are that the concentrations of MMP-2 and TIMP-1 (non-leukocyte derived components) are alike in the distal and proximal parts of the CMP whereas MMP-8 and MMP-9 (leukocyte derived components) are mainly represented in the distal part together with IL-8. Furthermore, MMP-8, MMP-9 and IL-8 show a several fold increase in CMPs from preterm deliveries.

The strength of the study lies in the procedures used for collection of both the CMP-dist aliquots and the whole CMPs. This ensures that we examine the CMP and not e.g. cervicovaginal fluid, which previously has been described in several publications [16,17]. Furthermore, the extraction protocol was designed specifically for CMP MMP [14,15]. Weaknesses are that the non-pregnant women had hormonal treatment and that the early pregnant CMP-dists were contaminated by 9% blood. However, the MMP and TIMP concentrations in blood from pregnant women are extremely low [18-20] and could therefore not have affected the results. Also, the quite low number of preterm CMPs included did not allow us to compare preterm and very preterm deliveries.

The high MMP-2 concentrations found in early pregnancy compared to the non pregnant state might reflect the non-leukocyte dependent phase of cervical remodeling that occurs at this gestational age [21]. It agrees with the immunohistochemical localization of MMP-2 shifting from stromal in the non-pregnant cervix to surface epithelial in the early pregnant cervix [22]. MMP-2 may also serve other functions in the CMP such as an activator of cytokines and anti-microbial peptides [9] or a mediator of the proteolytic processing necessary for unfolding of newly secreted mucins, which determine the rheological properties of the CMP [23].

The concentrations of MMP-8 and MMP-9 in amniotic fluid are independent of advancing gestational age but upregulated during normal spontaneous labor at term [13,24]. The same pattern is valid for CMP-dist MMP-8, MMP-9, and IL-8 which, compared to early pregnancy, showed a doubling at term but without reaching statistical significance. One might have expected that the cervical ripening process initiated weeks before labor would be reflected in the CMP-dists as significantly elevated MMP-8, MMP-9, and IL-8 concentrations at term [25]. Such an elevation was confirmed for IL-8 by subsequent *t-tests *of the early versus the term pregnant CMP-dists.

The lack of a clear evidence of an elevation of all three components as expected may be explained by the intense physiological inflammation detected in the CMP-dist compartment compared to the whole CMPs as described below: the effect of the progressive cervical maturation at term on CMP MMP-8, MMP-9, and IL-8 could be masked by the overall inflammatory state of the CMP-dist.

Furthermore, MMP-activity in the CMP-dist may be much better expressed by the dramatic decrease in the molar TIMP/MMP ratio from early pregnancy to term. However, TIMP-1 has many diverse effects other than MMP-inhibition including growth factor activity, effects on cell morphology, inhibition of angiogenesis, and anti-inflammatory effects that could play a role in the CMP [26,27].

The uneven distribution of MMPs in the CMP is striking with the upregulation of MMP-8, MMP-9 and IL-8, but not MMP-2 and TIMP-1, found in the CMP-dists compared to whole CMPs. Assuming that MMP-8 and MMP-9 are secreted by leukocytes [28], this biochemical compartmentalization confirms histological studies showing bacteria and numerous inflammatory cells, mostly neutrophil leukocytes, in the distal part of whole CMPs [29]. Cell-counting performed on suspensions of whole CMPs has previously verified that the CMP predominantly contains neutrophil leukocytes [15]. It is therefore highly probable that the inflammatory process, as reflected by the elevated concentrations of MMP-8, MMP-9 and IL-8 in the CMP-dists, protects the uterine cavity against ascending infection.

Intra-amniotic infection and preterm delivery are associated with high MMP-8 and MMP-9 concentrations in amniotic fluid [13,24] and in the fetal membranes [30]. In accordance with this, MMP-8, MMP-9 and IL-8 are increased in preterm whole CMPs. Furthermore, the only very preterm CMP included in the study displayed very high concentrations of MMP-8 and MMP-9 even though neither the mother nor the newborn showed signs of an infection. The clinical significance of this finding might be that increased MMP-8 in the CMP indicates an increased *risk *of intrauterine infection, whereas significantly elevated levels in the amniotic fluid indicate the presence of an *actual *infection.

The whole preterm CMPs also differed from the whole term CMPs in their doubled concentrations of MMP-2 and TIMP-1. Even though this difference did not reach statistical significance, the risk of a type two error should be considered: MMP-2 and TIMP-1 concentrations may have shown a significant increase in the preterm CMPs if more than four samples had been included.

A predisposition to ascending infection may be influenced by individual differences in the content of antimicrobial peptides in the CMP. With impaired innate and/or adaptive immune functions in the cervical canal during pregnancy, the susceptibility to ascending infection most likely is increased. One could hypothesize that in whole CMPs from preterm deliveries, not only the distal but also the proximal part of the CMP, is rich in leukocytes, MMP-8, MMP-9, and IL-8, thereby reflecting an ascending infection that has overcome the antimicrobial properties of the CMP.

## Conclusions

The CMP of pregnant women is highly differentiable from the CMP of non-pregnant women concerning the intensity of inflammation. In pregnancy, the CMP most likely hosts multiple neutrophil leukocytes as reflected by the very high concentrations of MMP-8, MMP-9 and IL-8. The inflammatory process is upregulated in the distal part of the CMP probably because the first job of the CMP is to protect the mother and the fetus against ascending infection. On the other hand, the non-leukocyte derived components MMP-2 and TIMP-1 are much more evenly distributed in the plug and may very well reflect cervical conditions. This could imply that in the CMP, the physiological and pathophysiological functions of MMP-2 and TIMP-1 differ from those of MMP-8 and MMP-9.

## Competing interests

The authors declare that they have no competing interests.

## Authors' contributions

NB, MH, CCD and NU have participated in the design of the study. NB carried out the experiments. NB, CCD and NU participated in the data analysis. The manuscript was written by NB and NU. CCD and MH assisted with revision. All authors have read and approved of the final manuscript.
